# Gene flow in commercial alfalfa (*Medicago sativa* subsp. *sativa* L.) seed production fields: Distance is the primary but not the sole influence on adventitious presence

**DOI:** 10.1371/journal.pone.0248746

**Published:** 2021-03-25

**Authors:** Sandya R. Kesoju, Matthew Kramer, Johanne Brunet, Stephanie L. Greene, Amelia Jordan, Ruth C. Martin

**Affiliations:** 1 Department of Agriculture, Columbia Basin College, Pasco, Washington, United States of America; 2 Statistics Group, Beltsville Agricultural Research Center, USDA Agricultural Research Service, Beltsville, Maryland, United States of America; 3 Vegetable Crops Research Unit, USDA, Agricultural Research Service, Madison, Wisconsin, United States of America; 4 Agricultural Genetic Resources Preservation Research Unit, USDA, Agricultural Research Service, Fort Collins, Colorado, United States of America; 5 Irrigated Agriculture Research and Extension Center, Washington State University, Prosser, Washington, United States of America; 6 Forage Seed and Cereal Research, USDA, Agricultural Research Service, Corvallis, Oregon, United States of America; University of Minnesota, UNITED STATES

## Abstract

In insect-pollinated crops, gene flow is affected by numerous factors including crop characteristics, mating system, life history, pollinators, and planting management practices. Previous studies have concentrated on the impact of distance between genetically engineered (GE) and conventional fields on adventitious presence (AP) which represents the unwanted presence of a GE gene. Variables other than distance, however, may affect AP. In addition, some AP is often present in the parent seed lots used to establish conventional fields. To identify variables that influence the proportion of AP in conventional alfalfa fields, we performed variable selection regression analyses. Analyses based on a sample-level and a field-level analysis gave similar, though not identical results. For the sample-level model, distance from the GE field explained 66% of the variance in AP, confirming its importance in affecting AP. The area of GE fields within the pollinator foraging range explained an additional 30% of the variation in AP in the model. The density of alfalfa leafcutting bee domiciles influenced AP in both models. To minimize AP in conventional alfalfa seed fields, management practices should focus on optimizing isolation distances while also considering the size of the GE pollen pool within the pollinator foraging range, and the foraging behavior of pollinators.

## Introduction

Since the introduction of genetically engineered (GE) crops, the acreage and the types of GE crops planted have been increasing. An important concern with the introduction of GE crops is the movement of GE genes to non-GE or conventional fields of the same crop or to wild populations of a close relative [1–7]. At issue is the occurrence of adventitious presence (AP), the unwanted presence of GE genes in non-GE products, which can negatively impact farmers that grow products for low AP tolerance markets, such as the export and organic markets. One strategy to limit AP in non-GE fields is to establish minimum isolation distances between GE and non-GE fields [7–14].

Alfalfa is one of the most important forage crops in the world and the third most valuable field crop in the United States [15]. Alfalfa is a perennial outcrossing plant that relies on insects for seed production. Pollinators must trip a flower, i.e. depress the keel of the flower in order to release its anthers and stigmas and pollinate a flower. Alfalfa seed producers use honey bees, *Apis mellifera* L., alfalfa leafcutting bees (ALCBs), *Megachile rotundata* F., and/or alkali bees, *Nomia melanderi* C., to pollinate alfalfa fields. Two GE alfalfa cultivars are commercially available in the United States and parts of Canada, the glyphosate resistant [Roundup Ready (RR)] cultivar and a low-lignin cultivar where the gene for low-lignin is stacked with the RR gene [16, 17]. With the release of GE alfalfa cultivars there exists an urgency to better understand the different factors that affect gene flow and AP in alfalfa seed production fields.

Pollinator-mediated gene flow occurs among alfalfa seed production fields [7, 8, 11, 18], thus it is important to understand the variables that influence the movement of GE genes. Previous studies have emphasized the importance of distance between GE and non-GE fields on pollen mediated gene flow [7–12, 18]. Besides physical distance, there are likely other variables that affect the probability of a gene flow event. For example, pollinator species, pollinator density, and pollinator foraging behavior can all affect gene flow [19–26]. Some insect pollinators move pollen longer distances and different bee species can differentially affect outcrossing rate and gene flow [24, 27, 28]. Previous studies in alfalfa suggest that honey bees carry pollen longer distances than ALCBs [8, 11, 18]. The foraging range of a bee can be affected by its body size and by the availability and distribution of resources over the landscape [29–31]. Larger bees tend to have greater foraging ranges [29] and a maximum foraging distance of 5.98 km has been observed for honeybees in alfalfa [12]. Pollinators minimize travel distances between flowers in habitats rich in floral resources and avoid florally poor areas [28, 31, 32]. Landscapes with high coverage and low fragmentation of semi-natural areas can decrease the foraging range of bees [31]. Plant density can influence gene flow by pollinators but not all pollinator species respond similarly to a change in plant density [24]. High plant density often decreases gene flow [33–35] by increasing the number of flowers visited in a patch and decreasing the number of patches visited by a pollinator in a foraging trip. Besides the density of the crop, greater pollinator density can increase gene flow potential as more bees are present for a limited resource increasing the chances of moving pollen to a different field [23]. Bee species also have differences in tripping rates, the proportion of visited flowers whose sexual organs are released when the pollinator applies weight on the flower, with greater gene flow associated with lower tripping rates [26].

Production practices, crop characteristics and environmental variables can also affect gene flow. The position of hives or bee boards in the field may affect movement of GE genes among fields with greater gene flow potential for hives located at the edges of a field. A bee board is a panel with cavities where cavity-nesting solitary bees can lay their eggs. The size of GE fields influences the amount of GE pollen in the vicinity and impacts the probability of a GE gene flow event [36]. Outcrossing crops, where pollen from a plant fertilizes other plants in the field, have a higher probability of gene flow relative to self-fertilizing crops, where pollen from the same plant is used for fertilization [37, 38]. Alfalfa plants tend to have a high outcrossing rate although variation among plants and studies has been reported [38]. Because perennial crops remain in the same field for more than a year and gene flow is likely to occur each year, perennial crops are considered to have a higher gene flow risk in an area relative to annual crop plants [37, 38]. Pollen viability, the length of time pollen remains viable after being picked up by a pollinator, will affect gene flow potential [39]. Environmental factors can affect flower density and duration, and pollinator behavior [20–22, 36, 40–43]. Low water availability decreases flower production which negatively impacts the resources provided by the plants to the bees [44, 45]. According to Scorza et al. [13], gene flow was correlated with distance and with weather conditions such as air temperature and rainfall. Different bee species are differentially affected by temperature and bees do not fly in the rain [46, 47]. While wind can affect the direction of gene flow in wind-pollinated crops [48, 49], no impact of wind on gene flow direction has been demonstrated in insect-pollinated crops [26].

Very few studies have examined how variables, other than distance, influence the occurrence of AP in alfalfa. This is especially true for studies that use commercial seed production fields at the landscape level. The alfalfa seed production industry in the United States currently relies only on isolation distances [7] to limit AP in official seed production areas that focus on either GE or AP-sensitive production (https://www.alfalfa.org/CSCoexistenceDocs.html). The industry relies on AP testing to manage AP levels in conventional seed. The development of a more flexible framework that considers multiple variables would give alfalfa seed producers more control over expanding and contracting isolation distances as they manage AP in conventional alfalfa seed production fields.

In this study, we examine how variables, including distance to the GE source, influence AP in alfalfa seed production fields. These variables include pollinators and different aspects of their management, field size, proximity to riparian and rangeland areas and various environmental and topographical factors. Results from this study provide a wider framework to understand gene flow in alfalfa seed production fields. This information can help growers develop more effective methods to reduce AP and facilitate the coexistence of the different alfalfa seed-production markets.

## Materials and methods

### Study fields, sampling design, and assessment of adventitious presence

**Fig 1:**
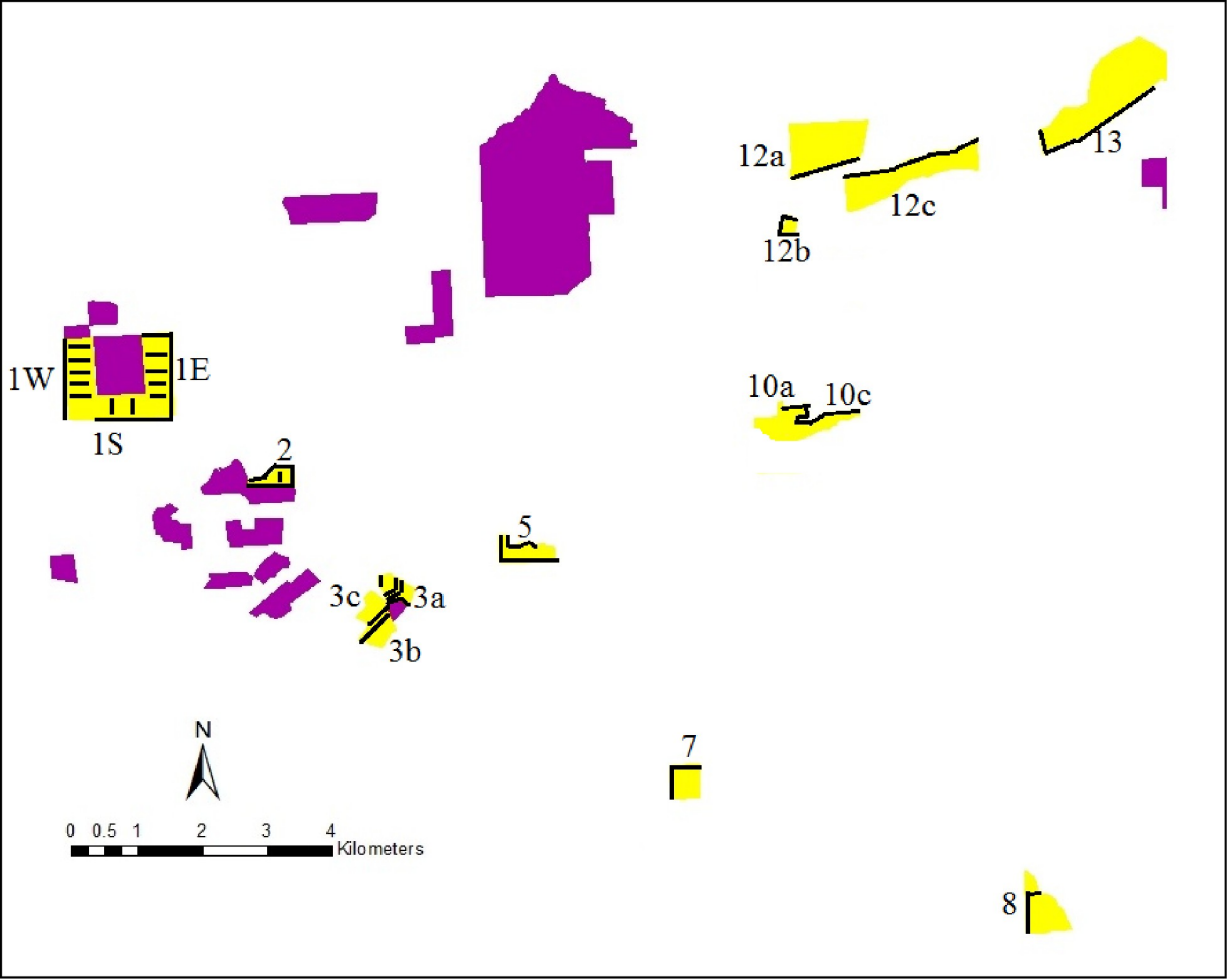
Distribution of commercial GE seed fields (pink) and conventional seed fields (yellow) that we sampled for GE gene presence in 2013 near Touchet Valley, Walla Walla County, Washington. Black lines represent transects and edges sampled.

Our study fields were located in the Touchet area, Walla Walla County, Washington. Study was carried out on private fields and owner of the fields gave verbal permission to conduct the study on their site. Details of the study area, sampling design and overall assessment of AP are described in Kesoju et al. [7]. Fourteen commercial conventional alfalfa seed production fields located at different distances from GE seed production fields were selected and sampled (Fig 1). One of the fields was planted with three different alfalfa varieties, and because each variety was spatially separated from the others, we included each variety as a separate field, which provided 16 fields for this study. The most distant conventional field was 11 km away from the nearest GE field [7]. In fields located approximately 250 m or more from a GE source, we focused on sampling field edges where gene flow rates can be higher than field centers [22]. The five study fields located < 250 m from a GE source were intensively sampled. In addition to sampling all four edges every 30 m, seed samples were obtained every 15 m from transects that went across the field (Fig 1). In this study, “sample” refers to seed collected at a specific distance in a specific field. For the 11 remaining fields, seeds were sampled every 30 m along one or two edges closest to a GE source field. Most samples were obtained directly from the seed harvest stream of the combine during harvest in September and October. Approximately 750 grams of seeds were obtained for each sample. In two fields (3b and 3c), farmers left plants at the edges of the fields for us to harvest by hand. We hand harvested pods along a 30 m stretch, and threshed each sample. The hand-collected samples were approximately the same size (700–750 grams) as samples collected from a combine.

To determine baseline levels of AP in conventional fields, 500 g samples of the original seed lot used to plant the field, which we termed ‘parent seed lot’ were obtained. At the end of the season, following seed processing of the fields, we also obtained a 500 g seed sample from each field that represented a harvested bulk field sample, since it was acquired after seed had been processed at the seed cleaning and conditioning facility. Finally, our harvested seed samples were cleaned and scarified.

### Testing for glyphosate resistance gene

The harvested seed samples, the parent seed lots and the bulk field seed lots were assessed for the presence of the enzyme CP4 5-enolpyruvylshikimate-3-phosphate synthase (EPSPS). This enzyme indicates the presence of glyphosate resistance. Seed testing was performed using a combination of the seedling germination assay [7, 50] and AgraStrip RUR TraitChek test strips [5]. We used preliminary data from three fields to estimate the seed sample size needed to quantify AP. Based on these data, 7200 seeds were tested per harvested seed sample and adjusted to germination [7]. The phenotypic assay (seedling germination assay) was used to identify putative glyphosate resistant seedlings, which were confirmed using TraitChek test strips. The test strip provides a qualitative threshold test based on CP4 EPSPS-specific antibodies coupled to a color reagent. The putative glyphosate resistant seedling was ground with 0.5 ml distilled water in a 1.5 ml Eppendorf tube. The slurry was stirred using a disposable stirrer, and the TraitChek test strip was placed in the tube. After 5 min, based on the presence or absence of a colored test line, samples were scored as either positive or negative for the presence of gene CP4 EPSPS [5, 50].

### Pollinators

Alfalfa leafcutting bees and alkali bees are used as managed pollinators of alfalfa in Walla Walla County. By county ordinance, honey bee hives are limited. Alfalfa leafcutting bees are solitary cavity nesting bees [51]. In alfalfa seed-production fields, bee boards placed in trailers or domiciles are used to provide nesting sites for ALCBs [51]. Female ALCBs will not forage or remain in an area unless they can nest and use the cavities to lay eggs [52]. Each egg is laid in a leaf collected by the bee and is provisioned with both pollen and nectar. Increasing the number of domiciles augments nesting cavities and helps maintain more female ALCBs in the field [52]. To ensure uniform pollination, seed producers in the Touchet area place ALCB domiciles at regular intervals (15 m apart), facing east, throughout the field in June. Alfalfa leafcutting bee cells, which contain bee larvae, are kept in cold storage over the winter and are incubated in early summer, so that bees emerge in synchrony with the alfalfa bloom.

Alkali bees are solitary bees but their nesting biology differs from ALCBs. Alkali bees are ground-nesting bees that prefer salty soils [53]. In order to cultivate these bees, farmers build bee beds with the right soil, salt, moisture conditions, and combinations of sun and shade. The Walla Walla Valley in Washington, is unique in that farmers use both alfalfa and the alkali bees. “Bee beds” are parcels of open soil to encourage female alkali bees to nest and raise their young, ensuring generations of pollinators and profitable seed yields. These beds have been maintained for over 50 years, underscoring the insect’s importance to local alfalfa growers. Alkali bees are efficient pollinators of alfalfa [54]. Alkali bee emergence begins as early as late May but will typically peak in mid- to late June [55], similar to that of the ALCBs. We observed nearly 30 alkali bee beds in the study area. The location of each ALCB domicile or alkali bee bed was recorded using a GPS. Both managed bee species typically forage for 4 to 6 weeks and foraging activity declines rapidly as July progresses.

To determine bee abundance in the fields, in early June 2013, pollinator surveys were conducted in the conventional alfalfa seed production fields. Pollinators were collected with a sweep net every 161 m, within a 3 m strip along the field edge. Sampling was done early in the morning when temperatures were less than 35 ^0^C and wind speed was below 6.71 m s^-1^. Five 180^0^ sweeps were used for each collection with the observer moving one-step forward between each sweep. Pollinators caught in the net were identified as alkali bee, ALCB, honey bee or native pollinators and these data provided the abundance for each of these four pollinator types in conventional alfalfa seed production fields.

### Explanatory variables that affect pollen-mediated gene flow

We examined various variables that could affect AP in alfalfa seed production fields (Table 1). We considered three kinds of variables in this study. First, are the variables inherent in the seeds planted such as parental AP. Second, we consider variables that might directly affect AP through pollinators such as pollinator abundance. Lastly, there are environmental factors such as slope that may indirectly affect the way pollinators forage. These can be divided into two categories: field-level and sample-level variables (Table 1). Field-level variables were characteristic of a field, and had a single value per field. Some examples of field-level variables were the direction of transects used in the conventional field in relation to the GE field; GE and conventional bees which represented the number of bees in the GE or conventional fields; ALCB stocking density and pollinator abundance measures (Table 1). In contrast, sample-level variables were measured multiple times within a given field and were characteristic of a given seed sample location within a field. These variables included, the distance from a seed sample collection site to the closest GE field center or GE field edge and the number of ALCB domiciles within a certain radius of the seed sample (Table 1).

**Table 1:** Explanatory variables used in the models to explain gene flow from commercial genetically engineered fields to conventional fields. The variables fell into two categories: a. Field-level and b. Sample-level variables. Field-level variables were characteristic of a field, and had a single value per field. Sample-level variables were measured multiple times within a given field and were characteristic of a given seed sample location within a field.

Variable	Description	Method of data collection	Transformations
**a. Field-level**			
Parent AP^a^	Presence of AP in parent seed lot	Parent seed used for planting and collected from growers and seed companies	logit(x)
Direction of transects in conventional fields in relation to the locations of GE^b^ fields	North, East, West, South, North-East, North-West, South-East, South-West	Collected by authors	No transformation
Bees in GE fields	1 = likely to encounter conventional field due to direction of domiciles, orientation of GE and conventional fields, wind direction, 2 = not likely to encounter conventional field	Collected by authors	No transformation
Bees in conventional fields	1 = likely to encounter GE field due to direction of domiciles, orientation of GE and conventional fields, wind direction, 2 = not likely to encounter GE field	Collected by authors	No transformation
Area of GE fields in 274 m circle zone (ha)	Circles of 274 m around each sample point were created. Area of the GE seed field fell in that circle were used	Collected by authors using buffer^d^ and clip^e^ methods	log(x)
Area of conventional fields in 274 m circle zone (ha)	Circles of 274 m around each sample point were created. Area of the conventional seed field fall in that circle were used	Collected by authors using buffer and clip methods	log(x)
Area of GE fields in 1.61 km circle zone (ha)	Circles of 1.61 km around each sample point were created. Area of the GE seed field fell in that circle were used	Collected by authors using buffer and clip methods	log(x)
Area of conventional fields in 1.61 km circle zone (ha)	Circles of 1.61 km around each sample point were created. Area of the conventional seed field fall in that circle were used	Collected by authors using buffer and clip methods	log(x)
Area of GE fields in 8.05 km circle zone (ha)	Circles of 8.05 km around each sample point were created. Area of the GE seed field fell in that circle were used	Collected by authors using buffer and clip methods	log(x)
Area of conventional fields in 8.05 km circle zone (ha)	Circles of 8.05 km around each sample point were created. Area of the conventional seed field fall in that circle were used	Collected by authors using buffer and clip methods	log(x)
Area of GE fields (ha)	Area of GE seed field in proximity to sample point was used	Information collected from growers	log(x)
Area of conventional fields (ha)	Area of each conventional seed field in proximity to sample point was used	Information collected from growers	log(x)
Area of the nearest alkali bee bed (ha)	Area of the nearest alkali bee bed to sample point (ha)	Information collected from growers	log(x)
Pollinator abundance (ALCBs^c^, alkali bees, honey bees, and native bees) in conventional fields	In summer 2013, conventional seed fields were surveyed and bees were collected along edges every 161 m	Authors collected data during the survey using a collection form	No transformation
Stocking density of bees (liters/field)	Gallons/acre were converted to liters/field	Information collected from growers	No transformation
**b. Sample-level**			
Distance of a sample from the edge of the nearest GE field (m)	Nearest neighbor distance was calculated from GE seed field to a sample point	Collected by authors using near^f^ method	log(x)
Distance of a sample from the center of the closest GE field (m)	Nearest neighbor distance was calculated from GE seed field to sample point	Collected by authors using near method	log(x)
Number of ALCB domiciles in GE fields	A circle of 274.32 m was created around each sample point in the conventional field. The number of GE domiciles within that circle was counted	Collected by authors using buffer and clip methods	sqrt(x)
Number of ALCB domiciles in conventional fields	A circle of 274.32 m was created around each sample point in the conventional field. The number of conventional domiciles within that circle was counted	Collected by authors using buffer and clip methods	sqrt(x)
Distance from ALCB domicile in GE field (m)	Nearest neighbor distance between a sample point and domicile in GE seed field	Collected by authors using near method	log(x)
Distance from ALCB domicile in conventional field (m)	Nearest neighbor distance between a sample point and domicile within the conventional seed field	Collected by authors using near method	log(x)
Elevation (m), Slope (deg), Aspect (deg)	30 x 30 m spatial resolution	USGS National Elevation Dataset (Gesch et al. 2002, Gesch 2007). Extract values to points^g^ method was used extract elevation, slope, and aspect values for sample point	log(x) for elevation and no transformation for slope and aspect
Distance from closest alkali bee bed (m)	Nearest neighbor distance from a sample point to the closest alkali bee bed	Collected by authors using near method	log(x)
Distance to riparian (m)	Nearest neighbor distance from a sample point to the closest stream	Collected by authors using near method	log(x)
Distance to open-range area (m)	Nearest neighbor distance from a sample point to the closest rangeland	Collected by authors using near method	log(x)
Distance from conventional field edge to sample point (m)	Nearest neighbor distance from a sample point to the nearest field edge	Collected by authors using near method	log(x)

^a^–genetically engineered.

^b^—adventitious presence.

^c^–alfalfa leaf cutting bees.

^d^–buffer method creates polygon/circle around the sample point.

^e^–clip method extracts area/domiciles of GE/conventional fields which fall in the buffers.

^f^–near method determines the distance from a sample point to the nearest input feature.

^g^–extract values to points method was used to extract the slope, aspect, and elevation values for each sample point.

^d-g^–ArcGIS 10.2 software was used for e-f methods.

For the environmental factors, average wind speed (m s^-1^), maximum wind speed (m s^-1^), maximum wind gust (m s^-1^) and wind direction (radians) were downloaded from AgWeatherNet (http://www.weather.wsu.edu/) and Weatherunderground (https://www.wunderground.com/). Data were collected for 22 weather stations located in Walla Walla County for the months when bees were actively pollinating commercial seed fields (June 1 to July 15). We could not obtain wind data at a sufficiently high resolution to use in our analyses due to a paucity of weather stations (only 2 stations) in the study area.

### Statistical analysis

Because the dependent variable and most explanatory variables were continuous, modeling was done in the multiple regression framework, using the R software [56]. Logit of the proportion AP in the seed lots was used as the dependent variable. Log (*p* / (1 –*p*)), where *p* is the proportion, have better statistical properties than proportions (or percent) for linear models. If the proportion was zero, it was replaced with a small random number (less than the smallest non-zero proportion) to avoid taking the log of zero. This is one of several strategies available to avoid taking logs of zero; for our analysis, it had the advantages of avoiding a spike in the data distribution from substituting a small constant value for zero and allowing the analysis in the usual regression framework rather than complicating it with additional dummy variables, or using less well-known and less understood methods. The correlations among all candidate independent variables on the original scale were examined and none were greater than 0.85. Since many of these variables were transformed (e.g. log transformed) to more evenly spread their values over their range, the correlations were further reduced. Independent variables and any transformation of them used in the analysis are given in Table 1.

As described above, some variables were sample-level, and others were field-level. Some categorical (qualitative) variables were included in the analysis (coded as dummy variables), see Table 1. We did two sets of analyses, one at the sample level, and one at the field level. The sample-level analysis, with 347 observations, included both sample-level and field-level variables and provided information about variables that affect AP level. For example, AP level could be affected by the number of bees in the conventional field, a field level variable, and by the distance from the conventional field edge to the seed sample point, a sample-level variable. Among fields, conventional fields with more bees could have less AP if the bees within a field trip many flowers, limiting the resources available and chances of tripping flowers to bees coming from another field. However, AP within a field can also depend on where the seed sample was collected, from the edge or further within the field, with a higher probability of AP expected at the edge of a field. Similarly, the distance from the closest alkali bee bed is a sample-level variable and the proportion of AP in a seed sample within a field could be greatest in samples collected closest to the nearest alkali bee bed. In contrast, a field-level analysis (16 fields) ignores within-field effects, and examines how differences among fields influence AP level.

For the sample level analysis, we used all candidate independent variables that were available at this level, but also initially included all variables only available at the field level. Results from early modeling attempts found that, given the number of field-level variables, these field-level variables combined to become a measure of field-to-field variability, rather than representing the true effect of that field-level variable. They were confounded with other field-level variables (measured or unmeasured), and signs and magnitudes of their coefficients changed depending on what other field-level variables were present in the model. Therefore, we dropped all field-level variables from the sample-level analysis and modeled field-to-field variability directly using 15 dummy variable orthogonal contrasts (contrasts with Field 10a, the first level as ordered by R) as additional candidate independent variables.

We used step-wise regression (function ‘step’ in R) for variable selection, using the lowest Bayesian information criterion to identify the ‘best’ model. The Bayesian information criterion is more conservative than Akaike information criterion for developing models, i.e. fewer explanatory variables are retained, and results are usually more robust. These are two of several information criteria, commonly used statistical tools that assess how related models compare in their fit to the same data set, balancing fit with the number of estimated parameters (see [57] for more details).

To determine if the model could be improved by accounting for residual spatial correlation, spatial models based on the residuals of predictive models using the nlme R package were explored [58]. The models examined included the predictor variables identified from the step-wise regression and a spatial autocorrelation parameter. A plot of the semi-variance against distance bins suggested that there was little spatial autocorrelation among residuals; an exponential model appeared acceptable for this residual spatial autocorrelation.

The relative importance of the regressors for the sample-level model (similar to a variance decomposition) was assessed using the R package, relaimp [59], which produces a decomposition of the explained variance into non-negative contributions; they can be interpreted as percent of the total variance. This is a good way to understand the explanatory ability of each of the independent variables in the model.

To analyze data at the field level (field-level analysis), we wanted to include sample-level independent variables, but needed to summarize them by field. We took means, following transformation (if any). The dependent variable was the mean of the sample level logits of AP, by field. We wanted to do a variable selection approach, as we did for the sample-level analysis, but could not use step-wise regression because the number of independent variables exceeded the number of fields. Lasso (least absolute shrinkage and selection operator) methodology using the R package, glmnet [60] was employed, which can be used in a ‘p > n’ scenario, shrinking the coefficients of candidate regressors that are not useful to zero. The retained variables based on minimizing lambda (note that standard errors and p values are not available for this method) were accepted.

## Results

### Adventitious presence in parent seed lots used for planting

Adventitious presence was detected in the parent seed lots used to plant the commercial conventional seed fields in this study. Ten out of 16 seed lots (65%) contained the glyphosate resistance gene, which was detected by the presence of the CP4 EPSPS protein (Table 2). With 7200 seeds tested per field, (500 g of seeds per field) an average of four seeds (0.05%) per field were found to have the glyphosate resistance gene (N = 16 fields).

**Table 2:** Field attributes and percentage of glyphosate resistance gene in parent seed lot, harvested bulk field sample and harvested seed sample from conventional field edges.

Field	Area of the nearest GE field (ha)	Area of conventional fields (ha)	Distance from nearest GE field (m)	AP in parent seed lot^1^ (%)	AP in harvested bulk field sample^2^ (%)	AP in samples harvested from edges^3^		
						Minimum (%)	Maximum (%)	Average (%)
Field1E	63.56	53.72	229	0.23	NA	0.15	4.89	1.42
Field2	42.06	13.00	113	0.11	NA	0.16	4.3	1.61
Field3a	5.13	15.65	156	0.06	0.23	0.01	1.26	0.54
Field1S	63.56	26.98	200	0.14	0.21	0.25	5.47	2.2
Field1W	69.94	53.72	239	0.03	0.33	0.05	3.83	1.64
Field3b	5.13	19.06	244	0	0.15	0.02	2.11	0.26
Field3c	5.13	11.08	259	0.11	0.15	0.04	0.81	0.31
Field13	78.08	109.52	1166	0	NA	0	0.11	0.02
Field5	5.13	21.18	1972	0.03	0.53	0	0.16	0.05
Field12b	435.12	4.09	2640	0	0.02	0	0.07	0.01
Field12a	435.12	36.44	2895	0	0	0	0.02	0.002
Field12c	78.08	67.08	3649	0.11	NA	0	0.18	0.02
Field10a	435.12	21.49	3795	0	0	0	0	0
Field10c	435.12	11.24	4308	0	0	0	0.03	0.003
Field7	5.13	20.47	5057	0.07	0.39	0	0.07	0.03
Field8	5.13	37.40	10805	0.02	NA	0	0.03	0.008

^1^Percentage of seeds with the glyphosate resistance gene in samples taken from parent seed lot.

^2^Percentage of seeds with the glyphosate resistance gene from in samples taken harvested bulk field lot.

^3^Percentage of seeds with the glyphosate resistance gene in samples taken from field edges.

### Adventitious presence in harvested bulk field seed

We obtained harvested bulk field sample during 2013 from seed companies for 11 out of 16 conventional seed fields we studied. Eight (73%) out of 11 of these seed lots had AP levels ranging from 0.02 to 0.53% (Table 2). From the 5888 seeds tested per field, an average of 11 seeds per field (bulk sample) were found to have the glyphosate resistance gene (N = 11 fields). In seven of the eight fields where we detected AP, the AP level was greater in the harvested bulk field seed samples compared to the parent seed lots used for planting (Table 2). Between field gene flow was evident for Fields 3b and 12b, since no AP was detected in the seed lots used to plant the fields, but harvested bulk field seed had 0.15 and 0.02% AP, respectively (Table 2). There was no evidence for between field gene flow in fields 10a, 10c and 12a, where no AP seeds were detected in either the seed lot used for planting or the harvested bulk field seed sample. In the other fields where AP was found in both the seed lot used for planting and the harvested bulk field seed sample, the likelihood of between field gene flow is high.

### Adventitious presence in the harvested conventional seed fields

We collected and tested a total of 229 samples along the edges of 16 conventional fields and detected the CP4 EPSPS gene in 176 samples (77%). On average, a seed sample contained 7567 ± 178 seeds of which 45 ± 6.25 seeds tested positive for the presence of CP4 EPSPS gene. Within a field, the average percentage AP ranged from 0.002 to 2.2% (Table 2 AP in samples harvested from edges). The AP levels detected in conventional fields located less than 260 m from a GE field were higher than AP levels found in conventional fields located 1000 m or more from a GE field (Table 2), suggesting a role of distance from GE field on AP.

We observed many differences between AP levels in the harvested bulk field seed sample (Table 2) and the seed samples we harvested from the field edges. In some cases, such as fields 1S and 1W, AP was much greater in the harvested seed samples relative to the bulk seed lot (Table 2). On average, 4440 and 5664 seeds sampled from the edges of the field were tested for CP4 EPSPS in fields 1S and 1W and 98 and 93 seeds respectively, were found to have the glyphosate resistance gene (Table 3). In other cases, for e.g. fields 5 and 7, there was a large decrease in AP in harvested seed samples relative to the bulk seed lot (Table 2). The AP level did not change much in other fields (fields 12a, 12b, 12c, and 10c). Of the 10,608 and 11,760 seeds tested in fields 10c and 12a, respectively, only an average 0.3 seed carried the glyphosate resistance gene (Table 3).

**Table 3:** Average number of seeds tested and average number of seeds testing positive for the glyphosate resistance gene in samples harvested from the edges of the fields.

Field	No. of samples harvested from the field edges	Average no. of seeds tested for CP4 EPSPS	Average no. of seeds testing positive for CP4 EPSPS	SE^a^ for seeds testing positive for CP4 EPSPS
Field1E	30	7418	130	44
Field2	21	5088	82	16
Field3a	29	6888	37	4
Field1S	10	4400	98	20
Field1W	19	5664	93	17
Field3b	22	6552	17	7
Field3c	13	6672	21	3
Field13	16	5592	1.3	0
Field5	10	11616	5	1
Field12b	8	9744	1	0
Field12a	7	11760	0.3	0.1
Field12c	11	8457	1	0
Field10a	7	10704	0	0
Field10c	10	10608	0.3	0.2
Field7	6	10752	3	0
Field8	10	11616	1	0

^a^ standard error.

**Fig 2:**
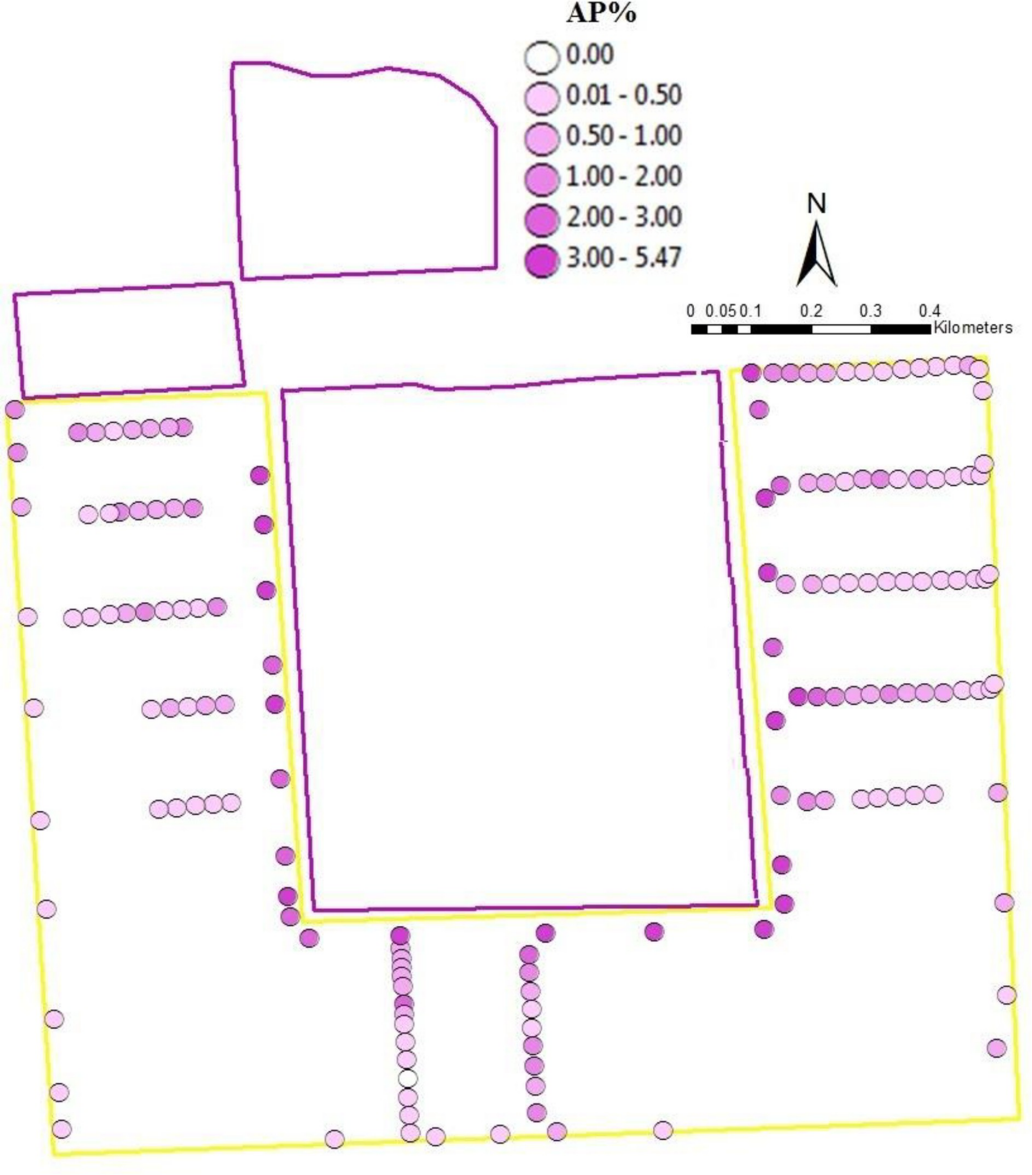
Genetically Engineered (GE) area comparable to conventional area. Adventitious presence (%) in conventional fields (1W, 1S, and 1E) at different distances from GE source field. Yellow outline color represents conventional seed field (right side–Field 1W; left side–Field 1E; bottom–Field 1S); purple outline color represents GE source field.

**Fig 3:**
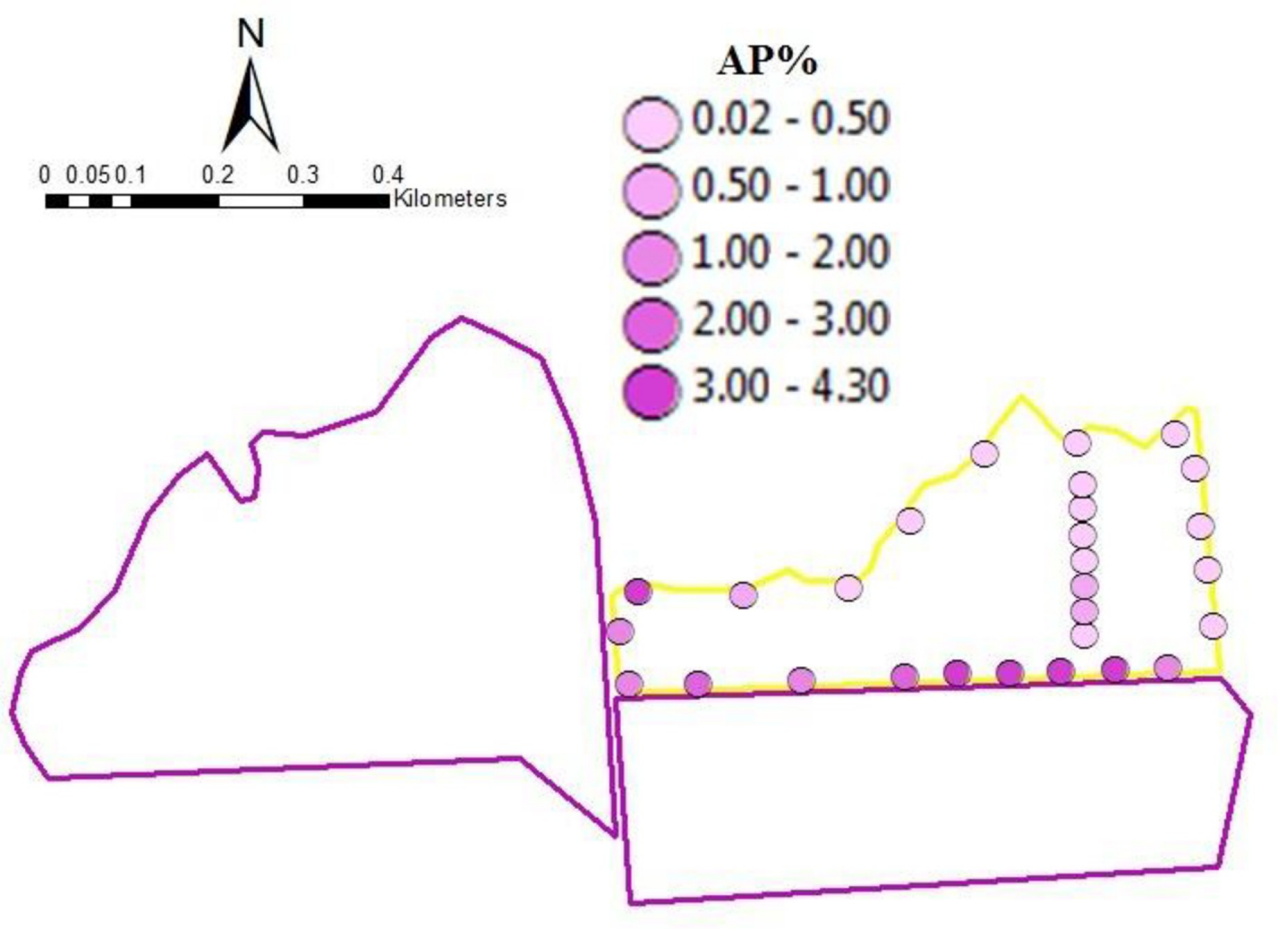
Genetically Engineered (GE) area greater than conventional area. Adventitious presence (%) in conventional field (Field 2) at different distances from GE source field. Yellow outline color represents conventional seed field; purple outline color represents GE source field.

**Fig 4:**
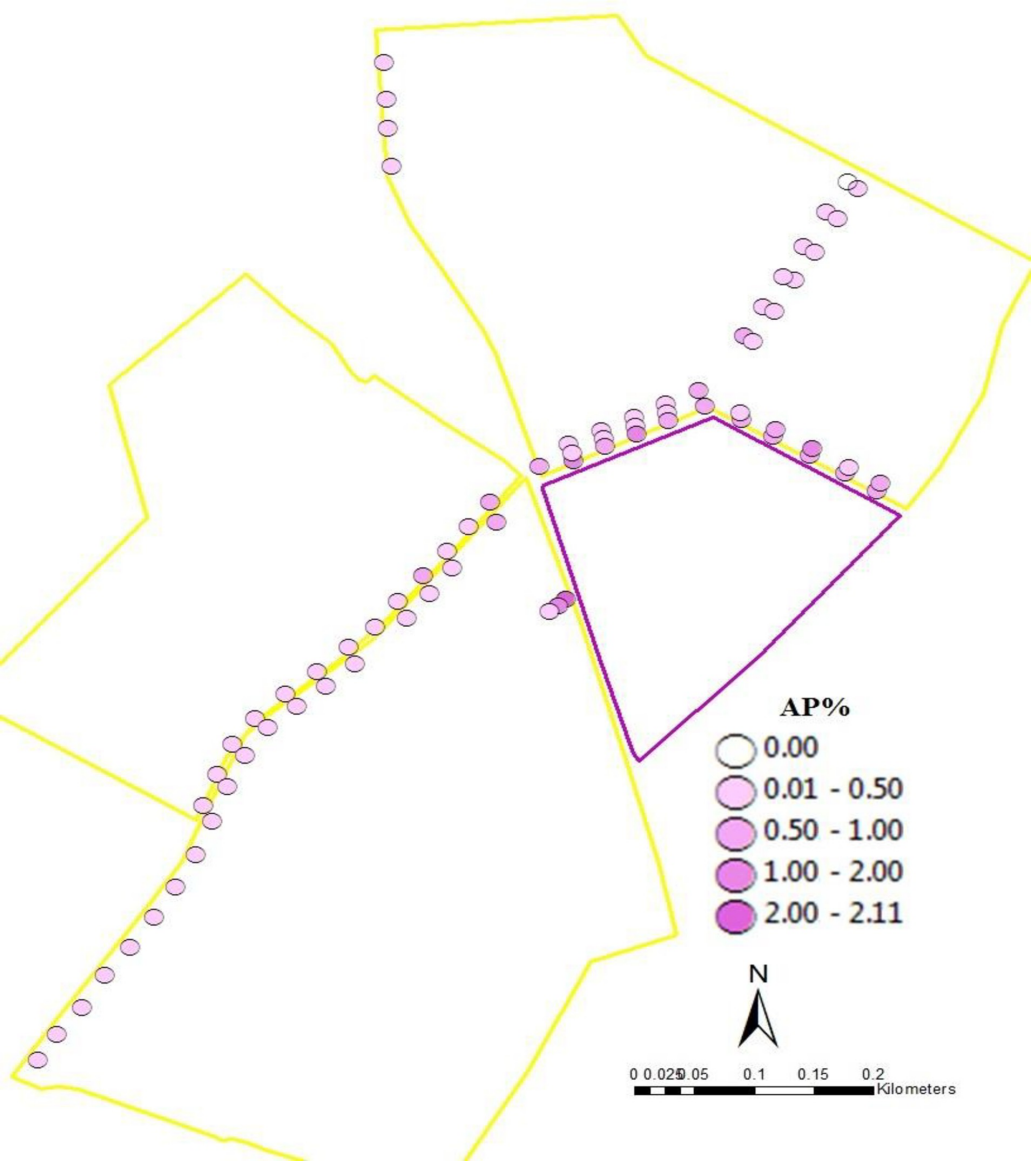
Genetically Engineered (GE) area less than conventional area. Adventitious presence (%) in fields 3a, 3b, and 3c at different distances from GE source field. Yellow outline color represents conventional seed field (North–Field 3a; SE–Field 3b; NE–Field 3c); purple outline color represents GE source field.

While our previous results only considered seed samples collected at the edges of fields, some fields (1W, 1E, 1S, 2, 3a, 3b, and 3c) were more intensely sampled by collecting seed samples in transects running throughout the fields. When these within-field samples were added to the 229 seed samples collected at the edges, we obtained a total of 347 seed samples. The glyphosate resistance gene was detected in 290 (84%) of these samples. The spatial arrangement of fields 1W, 1S, 1E, 2, 3a, 3b, and 3c allowed us to examine three cases where GE and conventional fields were located adjacent to each other but the relative areas of GE and conventional fields varied (Figs 2–4). In Fig 2, the GE area was comparable to the conventional area, in Fig 3, the GE area was greater than the conventional area and in Fig 4, the GE area was smaller than the conventional area. In all three situations, AP level dropped with increasing distances from the GE fields. The relative size of GE field affected AP, as only 1.15% GE seeds were found in the conventional field next to the smallest GE source relative to 3.32% and 2.63% for comparable or greater GE sources. This graphically confirms the results of the sample-level model, that distance from and area of a GE source affect AP.

### Variables affecting adventitious presence

Eleven variables were retained in the sample-level model to explain the percentage AP levels in the field samples (Table 4). The two distance measures together accounted for almost 66% of the explained variance, with distance from the closest GE field edge explaining 62%. This confirms that distance to the GE source is the major variable influencing the level of AP in alfalfa seed-production fields. As expected, gene flow decreased with increasing distance of the seed sample from the closest GE field center or field edge (negative estimate in Table 4).

**Table 4:** Variables explaining gene flow from commercial genetically engineered fields to conventional fields. Parameter estimates of explanatory variables from a stepwise regression procedure on logit transformed proportion of adventitious presence.

Variable	Transformations	Estimate	Standard Error	p-value	Proportion of total model variance
Distance of the sample from closest GE field center (m)	log(x)	-0.824	0.127	4e-10	0.041
Distance of the sample from closest GE field edges (m)	log(x)	-1.021	0.077	< 2e-16	0.615
Number of ALCB domiciles in conventional fields	sqrt(x)	-0.234	0.074	0.00175	0.004
Area GE seed fields within 1.61 km (ha)	log(x)	0.429	0.097	1e-05	0.296
Field (Field-to-field variation, 6 contrasts (6 d.f.)	Contrasts			All < 0.003	0.045

Besides distance between conventional fields and closest edge of a GE field, the next most important parameter that affected AP was the area of GE seed fields within 1.61 km of conventional fields, which explained 29.6% of the variance in AP in the model (Table 4). The larger the GE seed field area within a 1.6 km buffer around sample points, the greater the GE pollen pool. A third significant parameter was the number of ALCB domiciles in conventional fields, which explained 0.4% of the variance in AP (Table 4). The negative coefficient for the number of ALCB domiciles in the conventional fields indicated that the greater the number of ALCB domiciles in conventional fields, the lower the AP level was in these fields. Finally, six of the 15 field-to-field contrasts (field-to-field variation that represented other field-level differences between pollinator variables or other effects) together explained the remaining 4.5% of the variance in AP in the model (Table 4). Although significant, field-to-field differences were less important than distance and relative area of neighboring GE fields in explaining the level of AP in conventional seed fields. We found little spatial autocorrelation of residuals (too small to affect estimates or conclusions); the exponential spatial parameter estimate was about 1/100th of σ^2^, and not statistically significant (p = 0.11 using a likelihood ratio test).

### Field-level model

The Lasso coefficients point out how distance variables from the closest GE field (negative loadings) and area of GE field (positive loading) impact AP (Table 5). It also indicates a positive loading for numbers of ALCB domiciles in conventional fields (Table 5). In contrast to the sample-level model, the field-level model included the area of non-GE fields within 1.61 km, with a negative loading, and distance from nearest stream with a positive loading (Table 5).

**Table 5:** Field-level variables explaining gene flow from commercial genetically engineered fields to conventional fields. Lasso coefficients of variables from a lasso methodology transformed proportion of adventitious presence.

Variable	Lasso coefficients
Mean distance of the sample from closest GE field center (m)	-0.35
Mean distance of the sample from closest GE field edges (m)	-0.62
Mean number of ALCB domiciles in conventional fields	0.07
Mean area GE seed fields within 1.61 km (ha)	0.62
Mean area non-GE seed fields within 1.61 km (ha)	-0.17
Mean distance to riparian (m)	0.03

In summary, the same variables found to be important in the sample-level model (within- and between-field effects) were found to be important in the field-level model, and these were largely the ‘second kind’ of variables described above, variables that directly affect AP through pollinators.

## Discussion

Distance from GE fields strongly influences AP in conventional fields although other factors, such as the area of GE fields in the vicinity, also played a role. Decreases in AP with increasing distances between GE and non-GE test plots have been reported in previous gene flow studies in alfalfa [7–10, 18]. Similar declines in AP with increasing distances from the GE source have also been found in crops and fruit trees [13, 23, 42, 49, 61–63]. A reduction in gene flow at increasing distances is a common feature of wild plant populations and agricultural crops are no exception [64].

A greater GE field area reflects a larger GE pollen pool, which increases the probability of detecting AP in the conventional fields [36]. The GE field area within 1.61 km of the seed sample location explained close to 30% of the variance in AP in the model based on the sample-level analysis. The GE field area variable was also retained in the models derived from the field-level analyses. Moreover, the area of conventional (non-GE) fields within 1.61 km of the seed sample location was retained in the field-level model based on the Lasso analysis. Thus, conventional fields surrounded by a large area of GE fields have more AP. Moreover, conventional fields surrounded by large area of conventional fields have less AP. Therefore, the pollen pool surrounding the conventional field matters and affects AP. This finding is further supported in this study where the relative sizes of GE and conventional fields varied. More AP was detected in conventional fields adjacent to larger relative GE area. We expect the GE field area to have a similar impact on AP in other insect-pollinated crops. Such a pattern has been detected in cotton where the area of Bt cotton fields within 750m of the target fields best explained AP [23].

Interestingly, the GE area within 274 m or within 8 km of the seed sample location did not affect AP. These distances reflect specific isolation distances recommended for GE alfalfa seed production for distinct pollinators. For ALCBs, an isolation distance of 274 m is recommended; 1.6 km for alkali bees and 4.8 km for honey bees [65]. Moreover, the Association of Official Seed Certifying Agencies (AOSCA) Alfalfa Seed Stewardship Program (ASSP) specifies isolation distance of 8 km for an Identity Preserved Certificate for AP sensitive seed lots [7]. These data are based on differences in the distances at which GE genes were recovered following pollination by each of these bee species (summarized in Kesoju et al. [7]). In this study, ALCBs were present in all fields and were always the most abundant pollinator in every field. For the ten fields where bee abundance data were collected, alkali and honey bees were reported in about half of the fields and were less abundant than ALCBs in all fields. All three bee species were only reported together in three fields, and more generally either alkali bee or honey bee co-occurred with ALCBs. The importance of GE field area within a 1.6 km circle zone of the seed sample location indicated that one should not simply consider the foraging distance of the most abundant bee, here the ALCB, when measuring GE field availability. The alkali bee and ALCB are both very good trippers of alfalfa flowers [66] and the GE field availability and GE pollen pool that affected AP were within the foraging range of both ALCB and alkali bee.

The parent seed lots used to plant the field had levels of AP ranging from 0.00 to 0.23%, a range similar to the ones found for maize and cotton [23, 67, 68]. To our knowledge, this is the first study to detect AP in conventional alfalfa seed lots used to plant conventional seed production fields. The presence of GE genes in seed lots could negatively impact alfalfa seed producers who want to maintain GE-free fields and varieties. Although concerning, the presence of AP in the parental seed stock did not influence the level of AP occurring in seed harvested from planted fields. However, we recommend screening parental seed stocks for AP, especially if the intention is to produce conventional seed suitable for organic or export markets [69].

The number of ALCB domiciles in conventional fields was the only pollinator management variable retained in the sample-level model and it only explained 0.4% of the variance in AP. However, we observed little variation in pollinator management practices among fields within our study area. This makes sense as farmers try to optimize pollination strategies to maximize yield. Therefore, the analysis did not reveal an impact of distinct pollinator management practices on AP. Experimental manipulations of traits of interest or large-scale studies from areas where different management practices are used would better address these questions. In alfalfa seed-production fields and also in canola, cotton, fruit trees and most vegetables for seed production, bees move the pollen between flowers, plants and fields and in the process carry the GE genes [26]. The distance to the GE source impacts the ability of the distinct bee species to move genes a given distance [10, 16, 23]. The 1.6 km buffer zone used in this study reflects the foraging range of distinct bee species. It is thus important to remember that, although distance to GE source and area of GE source probably play important roles in explaining AP in other bee-pollinated crops, the scale that this should be examined at, should coincide with the foraging range of the specific pollinator(s) present, as was detected in cotton [23].

The number of ALCB domiciles in the conventional field was the only management practice that affected AP and the only variable whose impact varied between the sample-level and the field-level models. In the sample level analysis, an increase in ALCB domiciles decreased AP. In other words, within a field, areas with more ALCB domiciles are predicted to have lower levels of AP. Alfalfa leafcutting bee females need nesting sites to remain in the field and they tend to forage in the proximity of their nests [70, 71]. With more domiciles, one expects more ALCBs individuals foraging in the area, more flowers tripped and fewer floral resources available for bees coming in from surrounding fields. Thus, we expect bees coming from surrounding GE fields to select fields with fewer ALCB domiciles as they offer more resources. Because these bees may carry GE pollen, we expect higher AP in areas with fewer ALCB domiciles.

In contrast to the pattern observed within fields, the number of ALCB domiciles had a positive coefficient in the field-level model. Thus, among fields, we expect more AP in areas with more ALCB domiciles. Given the high cost of ALCBs, farmers are likely to place more domiciles only in fields with more flowers where this should increase yield. Fields with more flowers are more attractive to bees. We thus expect bees coming from other fields to be more attracted to fields with higher flower density. Having more bees coming into the field from GE fields would increase AP. Under such circumstances, fields with more ALCB domiciles would be expected to have higher AP. Thus, bee behavior influences AP levels in alfalfa seed production fields.

## Conclusions

The ecological patterns underlying gene flow in this study, such as the distance from the GE field, the size of the GE field and pollinator behavior, could apply to related seed production systems, particularly for other insect-pollinated crops. In settings where seed purity is desirable, seed producers and policy makers should consider 1) promoting the screening of parental seed lots for AP presence, 2) ensuring adequate isolation distance between GE and conventional fields, 3) monitoring the agricultural landscape to limit the GE area in proximity to conventional fields and 4) promoting a better understanding of pollinator behavior in order to limit pollinator movements between GE and conventional fields.

## Supporting information

S1 File (XLSX)
